# Linking susceptibility genes and pathogenesis mechanisms using mouse models of systemic lupus erythematosus

**DOI:** 10.1242/dmm.016451

**Published:** 2014-09

**Authors:** Steve P. Crampton, Peter A. Morawski, Silvia Bolland

**Affiliations:** Laboratory of Immunogenetics, National Institute of Allergic and Infectious Diseases, National Institutes of Health, Rockville, MD 20852, USA

**Keywords:** Lupus, SLE, Human genetics, Mouse models, Susceptibility genes

## Abstract

Systemic lupus erythematosus (SLE) represents a challenging autoimmune disease from a clinical perspective because of its varied forms of presentation. Although broad-spectrum steroids remain the standard treatment for SLE, they have many side effects and only provide temporary relief from the symptoms of the disease. Thus, gaining a deeper understanding of the genetic traits and biological pathways that confer susceptibility to SLE will help in the design of more targeted and effective therapeutics. Both human genome-wide association studies (GWAS) and investigations using a variety of mouse models of SLE have been valuable for the identification of the genes and pathways involved in pathogenesis. In this Review, we link human susceptibility genes for SLE with biological pathways characterized in mouse models of lupus, and discuss how the mechanistic insights gained could advance drug discovery for the disease.

## Introduction

**Fig. 1.**
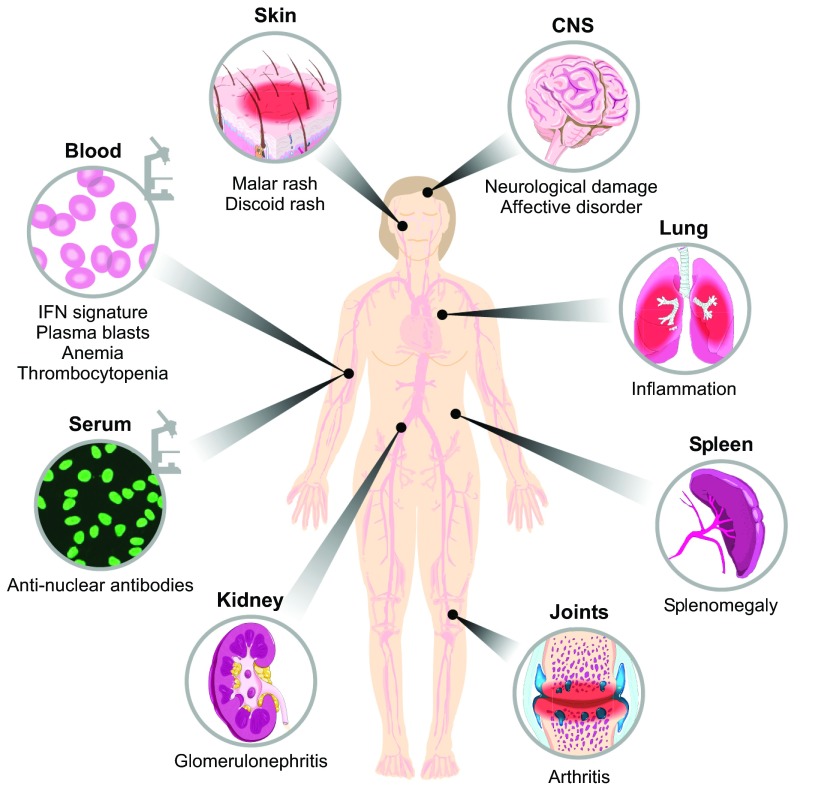
**Multi-organ involvement in systemic lupus erythematosus.** The characteristic appearance of antinuclear antibodies in the sera is a hallmark of lupus. Clinically, inflammation in lupus can affect the skin (rashes), CNS (neuropsychiatric disorders), lungs (serositis), joints (arthritis) and kidney (glomerulonephritis). Blood disorders such as anemia and thrombocytopenia can also be present. Hyperproliferation of immune populations can lead to an enlargement of the spleen (splenomegaly). A proper diagnosis of SLE requires four parameters linked to these phenotypes to be met, where at least one is clinical (e.g. the presence of a malar rash) and one is immunological (e.g. a positive for serum antinuclear antibodies).

Systemic lupus erythematosus (SLE; also known simply as ‘lupus’) is a multi-organ autoimmune disease characterized by loss of immunological tolerance, the system that normally protects self-components from attack by its own immune system. The major immunological targets (or autoantigens) are nuclear components of the cell, including double-stranded DNA (dsDNA), chromatin-associated proteins, and Ro (SSA), La (SSB) and Sm, the RNA-associated proteins that are most abundant in the nucleus (Rahman and Isenberg, 2008). Excess apoptotic debris containing abundant nuclear material is thought to be the source of these antigens. The self-reactive attack characteristic of SLE leads to a generalized activation of lymphocytes with concomitant production of autoreactive antibodies, plus an expansion of inflammatory cells that target multiple organs (Wakeland et al., 2001). Thus, the underlying cause of SLE seems to be inappropriate and chronic activation of the immune system, either as a consequence of inadequate clearance of immune targets or amplification of the level and duration of the immune response. Because this immune response is systemic and the targets are variable, the pathology of SLE can manifest in multiple organs with a variable course of disease, making it difficult to diagnose the disorder with accuracy. Indeed, the disease can involve the kidneys (nephritis), blood (thrombocytopenia and anemia; see Box 1 for a glossary of clinical terms), lung (serositis), the musculoskeleton (arthritis), skin (cutaneous rashes) and the central nervous system (psychosis and seizures) (Fig. 1) (Rahman and Isenberg, 2008).

**Box 1. Glossary of terms**

Arthralgia: joint pain.
Arthritis: a painful condition of inflammation and stiffness of the joints.
Complement: a group of proteins present in the blood plasma and tissue fluid that helps and complements the antibody response to clear pathogens from the body.
Creatinine: a compound that is produced by metabolism of creatine and excreted in the urine.
Cutaneous vasculitis: a condition characterized by inflammation of the wall of skin blood vessels associated with tissue necrosis.
Glomerulonephritis: a condition of acute inflammation of the kidney glomerulus, which is often induced by an immune response.
Hemolytic anemia: a form of anemia characterized by increased lysis of red blood cells and their subsequent removal from the blood circulation.
Hemophagocytic syndrome: a rare hematologic disorder characterized by over-production and activation of lymphocytes and macrophages.
Hypergammaglobulinemia: a medical condition associated with elevated levels of IgG in the blood sera.
Hyper IgM globulinemia: a medical condition associated with elevated levels of IgM in the blood sera.
Immune complex: a molecular complex that forms after the binding of an antibody to a soluble antigen.
Immunological tolerance: the failure to induce an immune response to an antigen. When defined as natural or ‘self’ tolerance, it refers to the failure to respond to the body’s own antigens.
Lymphoid hyperplasia: a rapid increase in the number of lymphocytes in the lymph nodes.
Lymphopenia: a condition associated with abnormally low levels of lymphocytes in the blood.
Myositis: inflammation and degeneration of muscle tissue.
Proteinuria: the presence of abnormal quantities of proteins in the urine, which can indicate damage to the kidneys.
Serositis: inflammation of the body serous tissues that line the lungs, heart, abdomen and inner abdominal organs.
Th1/Th2 responses: CD4^+^ T-cell-mediated immunity can be classified into two types of responses, depending on the profile of secreted cytokines. Type 1 or Th1 responses are generally induced by IFNγ and orchestrate cell-mediated immunity (e.g. enhanced phagocytosis). Type 2 or Th2 responses are commonly mediated by cytokines such as IL-4, IL-5 and IL-13, and drive humoral immunity (molecule-mediated immunity).
Thrombocytopenia: a deficiency of platelets in the blood.

The relatively high concordance rate between monozygotic twins in SLE indicates a genetic component to SLE susceptibility (Block, 2006; Deafen et al., 1992). A heavy gender bias is observed, with a typical female:male ratio of 10:1 (Beeson, 1994). This bias can be explained by a dosage effect of the X chromosome: the double X dose in females as well as in individuals with Klinefelter syndrome (associated with an XXY karyotype) increases SLE susceptibility tenfold (Scofield et al., 2008). However, the underlying molecular cause of this sex difference is not well understood; sex hormones might play a role in the female bias of SLE and it has been hypothesized that hormones alter B-cell responses that are important in autoantibody production (reviewed in Cohen-Solal et al., 2006; Grimaldi, 2006).

Although the onset of lupus is usually between the ages of 16 and 40, a particularly severe form of juvenile lupus can occur before the age of 16 (Bader-Meunier et al., 2003). As mentioned above, SLE can present with a variable course of pathology, and affected individuals frequently go into remission and experience sudden flares with unknown cause (Petri et al., 1991). Thus, SLE is clinically heterogeneous and the prognosis can be unpredictable. New insights into the genetics, endogenous or environmental triggers, and pathological mechanisms as well as the identification of markers of disease will be valuable in the design of novel treatments. Numerous mouse models of SLE have been developed and characterized to date, and they provide insight into the multigenic nature of genetic susceptibility to the disease. In this Review, we first discuss the clinical and immunological diagnostic features of SLE, and then provide an overview of some of the known immunological mechanisms underlying the disease. In the main body of the article, we describe some of the major mouse models of SLE that have been used to uncover genetic factors, and discuss how some of these findings have been linked to human genetic anomalies. Finally, we highlight how these research efforts have paved the way for prospective treatments for SLE.

## Human SLE: diagnosis of a complicated disease

Because of the overlap between the many possible symptoms of lupus and other disorders, SLE is difficult to diagnose accurately and, therefore, a complicated disease index system is used (Luijten et al., 2012; Yee et al., 2011). Within this system, at least four criteria must be met to make a diagnosis. One of the diagnostic markers needs to be clinical, and one immunological. Overall, diagnosis depends on histological evidence of lupus nephritis in addition to the presence of anti-nuclear or anti-dsDNA antibodies (Petri et al., 2012). The common immunological and pathological features that are used in the diagnosis of SLE are described below.

### Anti-nuclear antibodies

Anti-nuclear antibodies (ANAs) are highly prevalent and a major diagnostic marker for lupus. Serum autoantibodies appear as a consequence of systemic B-cell activation that is targeted toward self-specificities, the most common targets being nuclear proteins. IgM antibody isotypes are predominant in the serum of healthy individuals but, in SLE, immunoglobulin isotypes switch to the pathogenic IgGs, which activate inflammatory cells when deposited in tissues. Serum autoantibodies can be detected using an indirect immunofluorescence assay: Hep-2 cells are permeabilized and absorbed with patient sera, then detected with a fluorochrome-labeled secondary antibody. The autoantibody specificities are numerous; however, examples of autoantibodies that are observed before the clinical onset of the disease are: anti-dsDNA, anti-Ro, anti-La, anti-phospholipid, anti-Sm and anti-snRNP (Arbuckle et al., 2003). The presence of anti-dsDNA antibodies commonly correlates with active disease (ter Borg et al., 1990).

### Lupus nephritis

Lupus nephritis (glomerulonephritis), a key clinical feature of SLE, is thought to involve glomerular inflammation induced by immune complexes and complement deposition: first, immune complexes are formed by excess of antibodies that accumulate in the kidney; then, complement detects antibody aggregates and initiates an activation cascade that recruits monocytes and granulocytes to the site, ultimately causing tissue destruction. This type of nephritis is diagnosed by the presence of biomarkers for kidney damage such as proteinuria, creatinine and blood urea nitrogen (Reyes-Thomas et al., 2011).

### Cutaneous vasculitis and skin rashes

Cutaneous vasculitis and skin rashes are common in SLE. Typical manifestations are a Malar rash (acute), discoid rash (chronic), photosensitivity or oral ulcers (Kuhn and Landmann, 2014).

### Musculoskeletal inflammation

Musculoskeletal inflammation in SLE presents as arthralgias (joint pain), arthritis (inflammation of the joints) and myositis (inflammation of the muscles) (Cervera et al., 2003; Petri, 1995). This pathology can sometimes be a consequence of the heavy corticosteroid treatment (Petri, 1995).

### Hematological abnormalities

Hematological abnormalities, such as anemia, leukopenia, lymphopenia or thrombocytopenia, can be seen in various stages of lupus. These blood disorders can appear in the context of hemolytic anemia, hemophagocytic syndrome or can even be induced by treatment (Bashal, 2013).

### Neuropsychiatric lupus

Neuropsychiatric lupus can have multiple presentations, such as headaches, cognitive dysfunction or affective disorders (Brey et al., 2002). These symptoms are caused by an inflammatory pathology in the brain, in which autoantibodies have been proposed to play a dominant role (Abbott et al., 2003; Faust et al., 2010; Kowal et al., 2006; Lee et al., 2009a).

Generally, no single test can be used for the diagnosis of SLE. Instead, the presence of the symptoms shown above in various combinations would be used as a diagnostic tool. The more symptoms presented by an affected individual, the higher the score in the Systemic Lupus Erythematosus Disease Activity Index (SLEDAI) as defined by the American College of Rheumatology (https://www.rheumatology.org/Practice/Clinical/Indexes/Systemic_Lupus_Erythematosus_Disease_Activity_Index_SELENA_Modification/).

## An immunological anomaly underlies SLE pathology

**Fig. 2.**
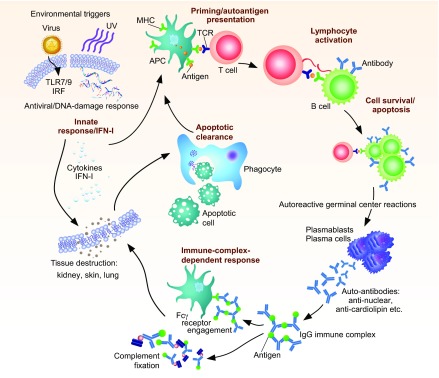
**SLE: the many players involved in systemic autoimmunity and tissue destruction.** Presentation of unknown antigens by MHC molecules leads to priming of CD4^+^ T cells. These cells then help B cells in autoreactive germinal centers undergo class switching, affinity maturation and differentiation into plasma cells that secrete high levels of soluble autoantibodies of the IgG isotype. These autoantibodies form immune complexes by binding autoantigens, and fix complement or engage Fcγ receptors on several different cell types. This can support inflammation and tissue destruction through the recruitment of inflammatory cells to tissues. Apoptotic cells from damaged tissues can be taken up by phagocytes, which present novel autoantigens, supporting further priming and autoreactivity. Engagement of TLRs by environmental triggers such as viral infection or DNA damage by UV rays contribute to the process by inducing the secretion of IFN-I and other cytokines, supporting lymphocyte autoreactivity as well as tissue destruction. APC, antigen-presenting cell; TCR, T-cell receptor. Bold text shows cellular functions that have lupus susceptibility genes related to them (see Fig. 3).

During the course of lupus disease, chronic immune activation usually precedes tissue injury (Fig. 2) (Mok and Lau, 2003). This immune dysregulation involves spontaneous loss of T- and B-cell tolerance to nuclear and other common self-antigens, and concomitant appearance of serum IgG autoantibodies (Li et al., 2007). The level of inflammatory cells is increased in response to IgG-induced cell activation or as a consequence of the increased level of circulating cytokines. Environmental factors, such as viral infection and UV light, together with spontaneous activation of innate pathways and/or nucleic acid sensors, can contribute to the high levels of cytokines, notably type I interferons (IFN-I) (Banchereau and Pascual, 2006) (Fig. 2). High serum IFN-I generates an ‘IFN signature’, a strong biomarker of autoimmune diseases that involves the elevated expression of hundreds of IFN-I-inducible genes (Baechler et al., 2003; Bennett et al., 2003). The IFN signature does not fluctuate in accordance with disease flares, suggesting that it might be secondary to the underlying cause of lupus (Landolt-Marticorena et al., 2009; Petri et al., 2009). However, elevated IFN-I-dependent cytokine and chemokine induction is considered as a biomarker of SLE disease activity (Bauer et al., 2006; Bauer et al., 2009; Kirou et al., 2005).

In the tissue injury phase of the disease, immune complexes generated from the circulating serum IgGs deposit into tissues such as the kidney and other vascularized organs where they activate the complement cascade or engage inflammatory cells via Fcγ receptors (Mok and Lau, 2003). This family of Fc receptors binds to the Fc portion of an IgG antibody that is bound to an antigen, leading to antigen internalization and consequent activation of the cell (Ravetch and Bolland, 2001). Immune complexes containing nucleic acids can also be internalized by plasmacytoid dendritic cells (pDCs) through Fc receptors to reach Toll-like receptors (TLRs) in the endosomes, leading to secretion of IFN-I and inflammatory cytokines (Means et al., 2005). This enhances local inflammation. Finally, defective clearance of apoptotic cell debris is thought to play a role, possibly by providing nuclear antigens that activate phagocytic cells, or nucleic acid components that activate innate pathways, further exacerbating inflammatory processes (Herrmann et al., 1998) (Fig. 2).

The following sections will assess the importance of these immunological pathways in the course of lupus disease by connecting insights into affected biological pathways from studies of well-characterized mouse models of SLE with what we have learnt from genetics studies of human SLE.

## Mouse models of SLE

Through many generations of inbreeding of various mouse lines, researchers have discovered strains that spontaneously develop SLE-like symptoms that resemble the human disease. Because of the polygenic nature of these models and the similarity of some histopathological features with human SLE, they have given insight into the genetic pathways that contribute to lupus susceptibility in humans. All models of SLE characterized so far have proven to be valuable, because studying a variety of mouse models of disease provides a broader view of the genetics and pathological mechanisms underlying autoimmunity. Some commonly used lupus-prone strains are discussed below.

### NZB/NZW F1 and mixed derivatives

Mice from the first generation of a cross between the strains New Zealand Black (NZB) and New Zealand White (NZW), known as NZB/NZW F1, develop a female gender-biased lupus-like syndrome characterized by lymphadenopathy, splenomegaly, elevated anti-nuclear antibody titers and immune-complex-mediated glomerulonephritis (Andrews et al., 1978; Helyer and Howie, 1963). Neither of the parent strains develops overt pathology, although NZB mice develop mild autoimmune hemolytic anemia (Sang et al., 2012). NZB/NZW F1 mice have an average lifespan of 10 months, and demonstrate the long-term presence of autoantibodies, thus representing a model of chronic lupus disease. Typical antibody specificities in NZB/NZW F1 mice include dsDNA, chromatin, histone H1, histone H2A, and ribonucleoproteins such as Ro, La and Sm, which are also characteristic of human SLE. In contrast with human SLE antibodies, those derived from NZB/NZW F1 mice lack the very common target specificities of the U1-snRNPs (Bender et al., 2014). This could reflect either the general heterogeneity in human outbred populations versus an inbred mouse strain or differential environmental factors encountered by the former.

To search for causal loci in this model of SLE, investigators backcrossed NZB/NZW F1 mice to NZW, then used brother-sister matings to generate 27 substrains, termed New Zealand mixed (NZM) mice (Rudofsky et al., 1993). Of these 27 substrains, NZM2410 was selected for further analysis because of the complete penetrance and severity of its pathology. Interestingly, the gender bias observed in parental NZB/NZW F1 mice disappears in the NZM2410 strain, perhaps because female aggravation is only a factor in mild forms of disease, which is also the case in humans, where the most severe cases in childhood lupus do not demonstrate gender bias (Bader-Meunier et al., 2003; Morel and Wakeland, 1998). The generation of congenic mice derived from NZM2410 has revealed the polygenic nature of the disease and linked different disease parameters to specific loci. For instance, B6.*Sle1*, a congenic strain on the C57BL/6 background containing chromosome 1 derived from NZM2410, develops autoantibodies against subnucleosomes, and displays spontaneous T-cell activation in the absence of renal disease (Mohan et al., 1998). A second congenic strain, B6.Sle2, derived from chromosome 4, displays lowered B-cell activation thresholds coincident with the appearance of polyclonal IgM in the sera, also in the absence of glomerulonephritis (Mohan et al., 1997). Interestingly, combining the two loci resulted in glomerulonephritis and enhanced mortality compared with the single congenic strains alone (Morel et al., 2000).

### MRL/*lpr*

MRL mice were derived from multiple crosses of inbred strains LG/J, C3H/Di, C57BL/6 and AKR/J (Andrews et al., 1978). A spontaneous mutation causing lymphoproliferation (*lpr* phenotype) occurred during inbreeding; this mutation was later identified as a retrotransposon insertion that disrupts the *Fas* gene (Adachi et al., 1993; Dixon et al., 1978), the gene that encodes the FAS death-inducing receptor essential to maintain an appropriate number of lymphocytes. MRL/*lpr* animals demonstrate B-cell hyperactivity, circulating immune complexes, lymphoid hyperplasia and glomerulonephritis (Andrews et al., 1978). Overall, MRL/*lpr* mice present with a severe form of disease. The inclusion of the *lpr* mutation in particular enhances disease severity, by triggering lymphoproliferative pathology. This model could also be used to provide insight into the more severe autoimmune lymphoproliferative syndrome (ALPS), which has been described in some cases in association with human SLE (Wu et al., 1996).

MRL/*lpr* mice produce a wide range of autoantibodies, including antibodies against DNA (Andrews et al., 1978), nucleosomes (Amoura et al., 1994), RNA polymerase (Stetler et al., 1985), cardiolipins (Gharavi et al., 1989), nucleolins (Hirata et al., 2000), phospholipids (Greenwood et al., 2002) and brain antigens (Moore et al., 1994). The pathogenicity of certain autoantibodies in the MRL/*lpr* mouse has been called into question. For instance, MRL/*lpr*-derived anti-DNA antibodies could not induce disease when injected into healthy control mice (Lake and Staines, 1986). MRL/*lpr* B cells normally secrete antibodies; however, a particular mutant was constructed that does not secrete antibody and still develops nephritis (Chan et al., 1999), suggesting that autoantibodies might not be responsible for this aspect of the disease. TLRs are also implicated in the initiation of disease in MRL/*lpr* mice, because a *TLR7*/*TLR9* double mutant protects the mouse from glomerulonephritis and lowers autoantibody production (Pawar et al., 2007).

Gender bias towards females is observed in some phenotypes displayed by the MRL/*lpr* model. Female mice exhibit higher serum IgG levels as well as increased ANA titers at 2–3 months of age, although this does not result in differences in overall systemic pathology or mortality (Andrews et al., 1978). More significantly, a bias towards female mice is seen in the neuropsychiatric component of SLE (Sakić et al., 1997).

Multiple cytokines have been linked to disease in MRL/*lpr* mice, including IFNγ (Haas et al., 1997; Santoro et al., 1983), IL-6 (Cash et al., 2010; Tang et al., 1991), IL-1β (Boswell et al., 1988; Lemay et al., 1996) and IL-18 (Esfandiari et al., 2001; Favilli et al., 2009). Regulatory or protective roles have been suggested for IL-10 (Yin et al., 2002) and IL-27 (Sugiyama et al., 2008). The humoral response in MRL/*lpr* mice is subject to regulation by IFN-I, which reduces antibody-mediated disease (Hron and Peng, 2004; Schwarting et al., 2005), whereas IL-21 produced by activated T cells drives autoantibody production (Herber et al., 2007). A number of the regulatory mechanisms involved remain unclear, and warrant further investigation using the MRL/*lpr* mouse model.

### BXSB.*Yaa*

BXSB mice demonstrate secondary lymphoid tissue hyperplasia, hypergammaglobulinemia, high titers of serum antiretroviral gp70 IgG, ANAs, and immune-complex-mediated glomerulonephritis, which is the primary cause of death in these mice (Murphy and Roths, 1979). The BXSB strain is derived from a C57BL/6 female and SB/Le male F1 backcrossed to SB/Le. Male progeny develop SLE with higher incidence, earlier onset and increased severity than females (Maibaum et al., 2000; Murphy and Roths, 1979). Given that the reciprocal cross (SB/Le female and C57BL/6 male) does not result in any gender bias of disease, this implies that the genetic disease-accelerating factor resides in the SB/Le Y chromosome. This genetic factor has been termed the Y-linked autoimmune accelerator, *Yaa* (Murphy and Roths, 1979).

Disease acceleration by the *Yaa* is genetically transferrable. NZW, MRL and *Sle1-3* lupus-susceptible strains all demonstrate exacerbated disease when they contain the BXSB Y chromosome (Hudgins et al., 1985; Merino et al., 1989; Morel et al., 2000). FcγRIIB-deficient mice, which also develop spontaneous SLE-like disease (Bolland and Ravetch, 2000), undergo a switch of autoantibody specificity from chromatin to nucleolar in the presence of the *Yaa* modifier (Bolland et al., 2002). The *Yaa* does not, however, induce autoimmunity on the C57BL/6 background. Thus, the *Yaa* genetic modifier is called an accelerator because by itself it does not initiate disease but rather it augments the severity in lupus-prone genetic backgrounds (Izui et al., 1988). Additionally, BXSB disease acceleration in male mice is not the result of hormone dysregulation (Eisenberg and Dixon, 1980). The *Yaa* is now known to be a 4-megabase translocation of the distal end of the X chromosome onto the pseudoautosomal region of the Y chromosome, which results in the duplication of over a dozen genes (Pisitkun et al., 2006). Among the duplicated genes is *Tlr7*, which is necessary and sufficient for *Yaa*-mediated disease acceleration: *Tlr7* deletion from the X chromosome abrogates *Yaa*-induced lupus phenotypes (Deane et al., 2007). TLR7 activation has been shown to affect antibody production by B cells, inflammatory production by monocytes and antigen presentation by dendritic cells. All these immune events together can explain the acceleration of disease observe with increased expression of TLR7 (Pisitkun et al., 2006).

### C57BL/6 derivatives: knockout and transgenic models

An alternative set of mouse models, beyond the naturally occurring lupus strains discussed above, consists of knockout or transgenic genetic modifications that profoundly affect a single susceptibility gene. Most of these mice have been generated in the C57BL/6 (B6) strain, which does not develop spontaneous disease but is permissive of disease induced by monogenetic alterations (Liu and Mohan, 2006). Thus, deficiencies in genes that prevent excessive lymphocyte activity or proliferation (i.e. those encoding FcγRIIB, Lyn, Fyn, CD22, PD-1, CD45 E613R, p21 and Bcl2 Tg) induce spontaneous disease in the permissive B6 strain. By contrast, sometimes the same mutation has an unremarkable or a completely different phenotype in the non-lupus-prone strain BALB/c (Bolland and Ravetch, 2000; Lawson et al., 2004; Nishimura et al., 2001). Some of these knockout mice, generated in the 129 strain and backcrossed to the B6 background strain, carry regions of the 129 genome surrounding the knockout locus. This can give an unreliable phenotype, because some 129/B6 mixed strains develop disease in the absence of genetic alterations (Bygrave et al., 2004). Nevertheless, the B6 strain is currently the most common laboratory strain used to characterize the effect of single-gene deletion, and a number of those gene knockouts develop SLE-like disease. We will discuss several of these strains in the context of specific molecular pathways in the sections below.

## Human genetics and lessons from mouse studies

**Fig. 3.**
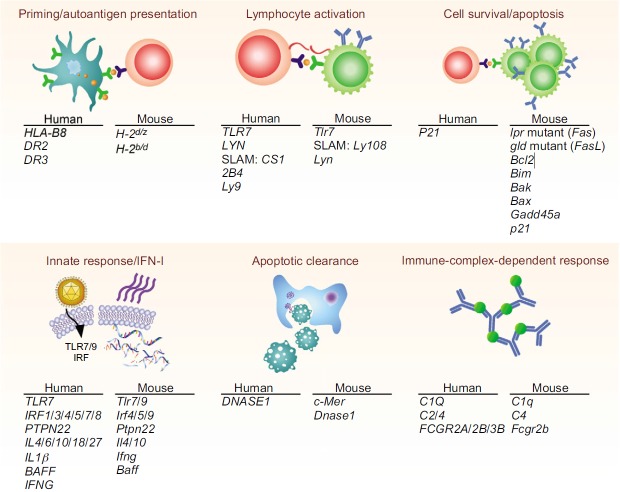
**Lupus susceptibility genes in humans and mice.** Genetic studies in humans and mice have found genes involved in different aspects of the disease. Human genes and their murine homologs are listed underneath headings that denote their function in the cell. See the main text for references.

Following completion of the human genome sequencing effort, various genome-wide association studies (GWAS) have been conducted on patient populations to pinpoint single-nucleotide polymorphisms (SNPs) associated with autoimmune disease (Lettre and Rioux, 2008). With these studies, various lupus susceptibility genes categorized by their effect on various biological processes within the lupus disease spectrum have been identified (Fig. 3). These genes can affect the activation of autoreactive lymphocytes by increasing antigen presentation of self-antigens, by lowering thresholds for activation, or by allowing survival of lymphocytes in suboptimal conditions. Another set of lupus susceptibility genes enhances innate responses that can occur spontaneously or as a consequence of environmental triggers. Clearance of apoptotic cells eliminates substrates of these innate pathways and deficiencies in these clearance mechanisms increase inflammatory responses. Finally, some susceptibility genes alter the way that immune cells are activated by IgG immune complexes and thus enhance tissue destruction. A combination of various susceptibility loci, in concert with environmental triggers, could contribute to the full expression of lupus pathogenesis (Wakeland et al., 2001). We will discuss several examples of human lupus susceptibility loci with supporting evidence in mice.

### *HLA* linkage in humans and MHC association in NZB/W mice

The human leukocyte antigen (*HLA*) region on chromosome 6 in humans and ‘histocompatibility 2′ (*H-2*) region on chromosome 17 in mice contain the highly polymorphic major histocompatibility complex (MHC) genes as well as other immunologically important genes (Browning and Krausa, 1996). Multiple susceptible haplotypes have been identified in various lupus patient cohorts, including *HLA-DRw2*, *HLA-DRw3*, *HL-A1*, *HLA-B8*, *HLA-DPB1*, *HLA-G*, *MSH5* and *HLA-DRB1* (Fernando et al., 2012; Ruiz-Narvaez et al., 2011; Taylor et al., 2011). The *HLA* region has the highest density of genes in the entire genome (Relle and Schwarting, 2012). This makes narrowing down lupus-susceptibility *HLA* haplotypes to disease-causing MHCs at the gene level particularly challenging because of significant linkage disequilibrium with surrounding genes (Wakeland et al., 2001).

In mice, the contribution of MHC haplotype to disease was first reported in the NZB/NZW F1 model (Babcock et al., 1989; Kotzin and Palmer, 1987). These studies implicated the *H-2^z^* locus derived from NZW mice as the main MHC haplotype contributing to this disease. However, the connection of this locus to disease seems to be somewhat complicated because transgenic expression of individual genes from the *H-2^z^* locus: *Ea^z^*/*Eb^z^* or *Aa^z^*/*Ab^z^* did not recapitulate disease in NZB (Rozzo et al., 1999; Vyse et al., 1998). Moreover, analysis of the MHC alleles derived from the C57BL/6 and C57BL/10 backgrounds (*H-2^b^*), in which the above-mentioned *E^z^* and *A^z^* transgenics were made, demonstrates that the *b* allele is linked very strongly to autoantibody production (that is, endogenous *H-2^b/d^*). This contribution of the *H-2^b^* allele to disease may explain why C57BL/6 is the most common background of lupus-prone knockout and transgenic models.

*HLA* risk haplotypes seem to be more strongly predictive of autoantibody specificity than disease pathogenesis. For example, individuals with the compound heterozygous risk alleles *HLA-DR2*/*HLA-DR3* are more likely to have autoantibodies against Ro, La and Sm, irrespective of an SLE diagnosis (Graham et al., 2007). Furthermore, when mice carrying a human transgene for *HLA-DR3* are immunized with the lupus autoantigen SmD, intermolecular epitope spreading occurs and anti-nuclear and anti-dsDNA autoantibodies appear in the serum (Jiang et al., 2010). This occurs in the absence of lupus nephritis. When human *HLA-DR2* and *HLA-DR3* transgenes are expressed in a lupus-prone NZM2410 background, *HLA-DR2* (and not *DR3*) accelerates the production of anti-dsDNA antibodies, but this has no effect on mortality (Paisansinsup et al., 2001). These data indicate that the specificity of lupus-related autoantibodies (determined by certain *HLA* haplotypes) that are present both in mice and humans are not necessarily the sole factors in determining end-organ pathogenesis.

### T-cell help component in lupus susceptibility: role of SLAM family members

The nine members of the SLAM (signaling lymphocyte activation molecule) family are receptors that regulate lymphocyte activation by coupling the effector SLAM-associated protein (SAP) to downstream signaling. *SAP*-knockout mice have a severe defect in humoral immunity that was found to be due solely to the lack of *SAP* expression in T cells (Crotty et al., 2003). Additionally, *SLAM*-deficient T cells fail to help B cells produce IgG antibodies *in vitro* (Yusuf et al., 2010). *SAP*-deficient mice were found to be protected from autoimmunity in a TLR7-induced model (Walsh et al., 2012). These results strongly implicate the SLAM-SAP axis in normal and autoreactive T-cell-dependent antibody responses. As mentioned earlier, congenic mice with the NZW-derived *Sle1* locus develop spontaneous T-cell activation and autoantibody formation (Mohan et al., 1998). The *Sle1* region encodes Ly108 (*slamf6*), another member of the SLAM family, which is implicated in autoimmunity in mice (Keszei et al., 2011; Kumar et al., 2006; Wandstrat et al., 2004). Different isoforms of Ly108 are expressed differentially in distinct strains of mice, and their relative ratio seems to affect B-cell tolerance and survival (Keszei et al., 2011; Wang et al., 2010).

Differential expression of SLAM family members is also observed in humans affected by SLE (Cunninghame Graham et al., 2008; Kim et al., 2010). Individuals with SLE have increased expression of CS1 (CRACC, CD319) on B cells and lower expression of 2B4 (CD244) on natural killer (NK) cells and monocytes compared with healthy controls (Kim et al., 2010). An SNP in the SAP-binding site of *Ly9* has also been identified in a cohort of SLE patients (Cunninghame Graham et al., 2008).

### Complement deficiencies are linked to lupus with high penetrance

The complement cascade consists of at least 25 proteins that ensure clearance of circulating immune complexes and apoptotic debris (Walport, 2001). It is long established that mutations in early components of the classical pathway strongly associate with lupus susceptibility in humans and mice (reviewed in Elkon and Santer, 2012). Patients with rare homozygous deficiencies in the gene encoding the first protein of the cascade, C1Q, develop a severe form of lupus in 90% of the observed cases (Bowness et al., 1994). Additionally, SNPs in *C1Q* have been associated with SLE in patient populations (Martens et al., 2009). Mice deficient in *C1q* also develop systemic autoimmunity, characterized by kidney disease caused by glomerular deposits of apoptotic bodies (Botto et al., 1998). Two other proteins in the early classical complement pathway, C2 and C4, are also associated with susceptibility to lupus (Arnett and Reveille, 1992). Complete loss of *C2* and *C4* is rare in humans, yet leads to a mild form of cutaneous lupus with some joint involvement (Schur, 1995; Yang et al., 2004). C4 deficiency also leads to lupus in mice, with 100% of the females developing antinuclear antibodies by 10 months of age (Chen et al., 2000). Defects in the complement cascade can have effects at the level of lymphocyte tolerance to nuclear antigens as well as the immune-complex-mediated tissue damage in affected organs. Complement factors seem to have a role in regulating B-cell activation and tolerance to self-antigens, and thus in autoantibody production, by engaging complement receptors in B cells (reviewed by Carroll, 1999).

### Inhibitory pathways prevent SLE

The inhibitory receptor for IgG, FcγRIIB, and the tyrosine kinase Lyn are important regulators of B-cell and myeloid-cell activation (DeFranco et al., 1998; Ravetch and Bolland, 2001). Deficiencies in these pathways result in enhanced humoral and inflammatory responses, thus contributing to lupus pathology.

FcγRIIB deficiency in mice results in spontaneous production of anti-nuclear autoantibodies, splenomegaly, anemia, glomerulonephritis and increased mortality (Bolland and Ravetch, 2000; Bolland et al., 2002). Manifestation of these features depends on the background strain, as the B6 background is permissive of lupus whereas the BALB/c background is resistant to lupus disease even bearing the same deletion of the *FcγRIIB* gene. FcγRIIB was found to be important in the regulation of autoantibodies, specifically in plasma cells (Fukuyama et al., 2005). Restoration of FcγRIIB levels on B cells in various lupus-prone strains was found to be sufficient to prevent autoimmunity (McGaha et al., 2005). FcγRIIb downregulation has been cited as a key phenotypic feature in the NZB/NZW F1 model of lupus (Rahman et al., 2007; Xiu et al., 2002). FcγRs in general were shown to be crucial for immune-complex-mediated kidney pathology in the NZB/NZW F1 model (Clynes et al., 1998).

In humans with SLE, memory B cells express lower levels of FcγRIIB (Mackay et al., 2006; Su et al., 2007). Moreover, an SNP in the gene encoding the receptor, FcγRIIB [Ile232Thr (I232T)], leads to a non-functional receptor and is strongly associated with SLE in populations from Asia and of Asian descent (Floto et al., 2005; Kono et al., 2005; Kyogoku et al., 2002; Lee et al., 2009b; Siriboonrit et al., 2003). In addition to *FCGR2B*, SNPs conferring altered functional proteins in the activating FcRs – *FCGR2A*, *FCGR3A* and *FCGR3B* – have also been discovered in association with SLE (Li et al., 2009). Owing to the high degree of linkage disequilibrium within this locus, it will be important to separate out the contributions of each of these mutations to susceptibility to disease (Kyogoku et al., 2002; Siriboonrit et al., 2003).

The tyrosine kinase Lyn is involved in negative regulation of B-cell signaling (DeFranco et al., 1998), and its deficiency in mice results in anti-dsDNA autoantibodies, splenomegaly, hyper IgM globulinemia and glomerulonephritis (Chan et al., 1997; Hibbs et al., 1995; Nishizumi et al., 1995). The kidney pathology in these mice is mild; however, it is enhanced in mice that are also deficient in Fyn, another Src-family kinase (Yu et al., 2001). The contribution of Fyn to kidney disease seems to be independent of T and B cells, suggesting that Lyn-dependent production of autoantibodies synergizes with Fyn deficiency in aggressive kidney pathology. The constitutive type 2 responses in *Lyn*^−/−^ mice also play a role in the lupus phenotype, because autoreactive IgE and basophils amplify glomerulonephritis (Charles et al., 2010). The Lyn-Th2 axis might be an important contributor to human disease as well. *LYN* SNPs are associated with SLE and lower expression of LYN protein and mRNA is observed in B cells isolated from lupus patients (Liossis et al., 2001; Lu et al., 2009). Interestingly, anti-dsDNA autoantibodies of the IgE isotype are elevated in active versus mild disease in SLE sera, suggesting a contribution of the type 2 response in active nephritis (Charles et al., 2010).

### Role of cytokines in lupus

Active SLE is characterized by high levels of chronic inflammation, indicated by elevated levels of inflammatory cytokines and chemokines. These factors might be useful for predicting disease activity and prognosis. Mouse models have been used to determine the role of these inflammatory proteins in disease development by applying crosses to specific knockout mice. Some of the results of these studies have been difficult to interpret, perhaps because of multiple effects of the same cytokine at different stages of disease. In the context of SLE, some of the best-studied cytokines are discussed below. The common occurrence of an IFN-I interferon gene expression signature in the peripheral blood of individuals with the disease strongly links type I interferons to SLE (Obermoser and Pascual, 2010). In line with the importance of IFN-I in the disease, treatment of melanoma or hepatitis patients with IFNα occasionally induces an SLE-like disease (Ho et al., 2008; Rönnblom et al., 1990). In mice, IFN-receptor deficiency ameliorates but does not completely eliminate lupus in several models of lupus (Richez et al., 2010; Santiago-Raber et al., 2003). Genes encoding the numerous IFN genes have not yet been found in genetic screens for lupus susceptibility factors but, as we will see in the sections below, multiple linkages have been found in molecular pathways of the innate immune response (IRFs, TLRs) that lead to IFN-I production.

Regarding type II interferons, some studies have demonstrated elevated *IFNG* mRNA levels and IFNγ targets in the blood and skin lesions from SLE patients (Carneiro et al., 2011; Karonitsch et al., 2009; Lit et al., 2007). Additionally, polymorphisms in non-coding introns of *IFNG*, which affect its expression, are associated with susceptibility to SLE and nephritis, with a stronger correlation when observed in conjunction with *IL18* SNPs (Hirankarn et al., 2009; Miyake et al., 2002; Tangwattanachuleeporn et al., 2007). Mice with increased expression of IFNγ, either through a transgene in the skin (Seery et al., 1997) or through deletion of a regulatory region (Hodge et al., 2014), develop SLE-like disease. In addition, ablation of IFNγ signaling provided protection against the disease in a T-cell-dependent model of lupus (Lee et al., 2012).

A gain-of-function SNP in the *IL4* promoter, originally identified in association with asthma (atopic disease), was positively associated with susceptibility to lupus (Rosenwasser et al., 1995; Wu et al., 2003; Yu et al., 2010). Forced expression of *IL4* under an MHC class I promoter precipitated a lupus-like disease with B-cell hyperactivity, anemia, anti-nuclear autoantibodies and kidney disease (Erb et al., 1997). Interestingly, this disease did not seem to require T cells, because ablating CD4^+^ T cells did little to alleviate the disease. This is in contrast to other lupus mouse models, which have a strict CD4^+^ T-helper-cell requirement for disease (Jevnikar et al., 1994; Koh et al., 1995; Walsh et al., 2012; Wofsy and Seaman, 1985).

Numerous studies have demonstrated higher circulating levels of IL10 in SLE sera and spontaneous production of the cytokine by B cells and monocytes (Csiszár et al., 2000; Gröndal et al., 2000; Houssiau et al., 1995; Llorente et al., 1993; Park et al., 1998). An SNP in the *IL10* locus is associated with SLE (Gateva et al., 2009). IL10 is generally an immunomodulatory cytokine that promotes Treg expansion (Moore et al., 2001), so it is uncertain whether elevated IL10 in SLE blood is a response to autoreactivity, or whether it is participating positively in the pathogenesis of the disease. In support of the first hypothesis, MRL/*lpr* mice deficient in *Il10* have a more accelerated and severe disease compared with *Il10*-sufficent mice (Yin et al., 2002).

### Nucleic-acid sensors are involved in autoantibody selection and aggravation of disease

Pathogen-derived nucleic acids are sensed by endosomal (i.e. TLR3, 7, 8, 9) or cytoplasmic (e.g. RIG-I–MDA5–STING) pathways. One potential outcome of engaging these DNA- and RNA-sensing receptors is IRF-dependent production of IFN-I (reviewed in Crampton and Bolland, 2013). Several lines of evidence implicate alterations in both antiviral pathways and cytosolic sensors of nucleic acids in the initial phases of autoimmunity.

Activation of endocytic TLRs, including TLR7 and TLR9, leads to IFN-I production by pDCs as well as the activation of B cells by simultaneous engagement of B-cell receptor and a TLR (Green and Marshak-Rothstein, 2011; Kadowaki et al., 2001). IFNα generated in response to self-RNA antigen is part of a positive feedback loop involving pDCs that supports increased TLR7 expression, thereby heightening responsiveness to self-RNA-containing immune complexes (Ganguly et al., 2009). Together, these two factors contribute to earlier onset and increased severity of lupus. Recent studies have revealed an increase in *TLR7* gene copy number as well as higher *TLR7* and *IFNα* mRNA levels in individuals with juvenile SLE (García-Ortiz et al., 2010). Similarly, expression of *TLR7* and *IFNα* are elevated in adults with SLE (Chauhan et al., 2013; Komatsuda et al., 2008; Wong et al., 2010). Genetic studies on human SLE patients also uncovered a number of *TLR7* polymorphisms associated with increased disease severity (Kawasaki et al., 2011; Wang et al., 2014), including one showing a *TLR7* SNP that leads to a pronounced IFN-I signature (Shen et al., 2010). Evidence from mouse models corroborates data obtained from human studies. Both the *Yaa*, derived from SB/Le mice, and *TLR7* transgenic models show an increase in the gene copy number and expression of *TLR7*, which results in a lupus-like disease marked by lymphocyte hyperactivation, ANAs and glomerulonephritis (Deane et al., 2007; Pisitkun et al., 2006). Additionally, genetic elimination of *TLR7* is sufficient to extinguish *Yaa*-induced disease (Deane et al., 2007). TLR9 recognizes dsDNA with a CpG motif and, unlike TLR7, it does not accelerate disease in murine lupus. TLR9 protects mice on the B6/*lpr* or MRL/*lpr* backgrounds; deletion of this receptor results in more severe clinical disease and earlier mortality (Christensen et al., 2006; Wu and Peng, 2006). Although TLR9 provides protection against lupus in mice, deleting it lowers anti-dsDNA antibody production (Chen et al., 2011). Additionally, several studies have suggested that humans with SLE display increased TLR9 expression (Chauhan et al., 2013; Komatsuda et al., 2008; Midgley et al., 2012). It is possible that TLR9 has multiple functions with opposing effects that can be uncoupled. It is also unclear whether anti-dsDNA antibodies are directly pathogenic or merely correlate with pathology but do not initiate harmful responses. More studies are required to uncover this dichotomy and to clarify the direct effect of ds-DNA.

The *IFIH1* gene, which encodes the RNA sensor MDA5, is a risk factor in several autoimmune diseases, including SLE (Cen et al., 2013; Gateva et al., 2009; Smyth et al., 2006). In mice, transgenic expression of MDA5 results in a chronic interferon signature and aggravated lupus disease (Crampton et al., 2012). Furthermore, mice expressing an MDA5 gain-of-function mutation develop IFN-I-dependent autoimmunity in the absence of an external trigger, presumably due to ligand-independent MDA5 activation (Funabiki et al., 2014).

Finally, loss-of-function mutations in the nucleic acid repair exonuclease TREX cause a severe immune-mediated neurodevelopmental disorder termed Aicardi-Goutieres syndrome, whereas mutations with a milder effect have been linked to chilblain lupus (Rice et al., 2007). TREX1 has been shown to be crucial in preventing autoimmunity in mice, because deficiency in this nuclease causes severe IFN-I-induced inflammatory pathology (Gall et al., 2012; Stetson et al., 2008). Thus, systems that eliminate altered forms of nucleic acids are essential to lower spontaneous activation of innate pathways, and might underlie the pathological consequences of UV exposure in SLE-sensitive genetic backgrounds.

### The interferon response factor (IRF) family includes multiple risk factors for lupus

The activity of the IRF transcription factors drives the production of IFN-I in both immune and non-immune cells (reviewed in Honda and Taniguchi, 2006). Multiple polymorphisms in *IRF5*, *IRF7* and *IRF8* have been identified as risk factors in SLE (Kawasaki et al., 2008; Li et al., 2014; Salloum et al., 2010; reviewed in Salloum and Niewold, 2011). IRF5 is crucial for dendritic-cell and B-cell development (Lien et al., 2010), whereas IRF7 acts downstream of endosomal TLRs and induces IFN-I. IRF7 expression correlates with an active IFN-I signature in some individuals with SLE (Sweeney, 2011). An additive effect of *IRF5* and *IRF7* polymorphisms on serum IFNα levels is seen in patients positive for anti-dsDNA antibodies (Salloum et al., 2010). In mice, IRF5 deficiency protects from disease in various models of disease, and it has been suggested that this IRF regulates monocyte expansion and Th2-biased IgG class switching (Feng et al., 2012; Richez et al., 2010; Yang et al., 2012). IRF8 seems to be important in differentiation of IL17-producing cells (Baccala et al., 2013; Ouyang et al., 2011; Schönheit et al., 2013) and the promotion of germinal center B cells (Shin et al., 2011).

### *PTPN22* susceptible alleles are common and span multiple autoimmune diseases

PTPN22 is a phosphatase that suppresses lymphocyte activation, and is a risk factor for SLE as well as other autoimmune diseases. The major *PTPN22* polymorphism, R620W, causes an increase in autoreactive B cells, and defective central and peripheral B-cell tolerance in humans (Harley et al., 2008; reviewed in Rhee and Veillette, 2012). A recent study showed that PTPN22 also functions in myeloid cells, where it augments TLR- and IRF-dependent IFN-I production (Wang et al., 2013). In the absence of PTPN22, or in a mouse expressing only the SLE risk allele *PTPN22W*, IFN-I responses are decreased in response to TLR3 and TLR9 ligands, and mice lose protection from induced arthritis and colitis. These data demonstrate that PTPN22-dependent IFN-I production is part of a protective mechanism. However, in human SLE, IFN-Is are largely associated with increased disease severity, and IFNα therapy in humans is linked to an induction of SLE-like disease (Ho et al., 2008; Rönnblom et al., 1990). In light of this new IFN-regulatory role for PTPN22, more studies are required to elucidate the mechanism by which this gene can regulate autoimmunity.

### Apoptotic signals and cell cycle checkpoints as intrinsic determinants in lupus

Perturbation of signals that normally determine programmed cell death (apoptosis) of B and T cells have the potential to promote autoimmunity. Mice expressing a human transgene encoding the pro-survival gene *BCL2* restricted to the B-cell lineage develop antinuclear autoantibodies and glomerulonephritis along with immune-complex deposition (Strasser et al., 1991). On the other hand, mice deficient in pro-apoptotic factors *BAX* and *BAK*, or *BIM*, develop a lethal systemic autoimmune disease (Bouillet et al., 1999; Mason et al., 2013). *BCL2* levels have been found to be elevated in circulating B cells and T cells as well as in the kidneys from humans with SLE (Fathi et al., 2006; Gatenby and Irvine, 1994; Graninger et al., 2000). In addition, serum levels of the B-cell-activating factor (BAFF; also known as BLyS), known to be crucial for B-cell survival, are elevated in individuals with lupus and correlate positively with the appearance of autoantibodies and disease activity indexes (Cheema et al., 2001; Chong et al., 2014). Transgenic mice that overexpress BLyS either ubiquitously or in the liver develop a lupus-like disease with B-cell hyperplasia, spontaneous germinal centers, autoantibodies to DNA and kidney disease (Khare et al., 2000; Mackay et al., 1999). The disease manifestations seem to be independent of T-cell help and dependent on signals by the MyD88 adaptor molecule, likely through TLR7 or TLR9 engagement (Groom et al., 2007).

Alterations in cell cycle checkpoints could lead to unrestricted division of cells with autoreactive potential and thus increase the risk of developing SLE. Deficiency of p21, an inhibitor of cyclin-dependent kinases that is essential for cell cycle arrest, leads to T-and B-cell autoimmunity and kidney disease (Balomenos et al., 2000). The effects of p21 are also amplified in the presence of a *Bcl2* transgene, underscoring the importance of both of these pathways in tolerance (Santiuste et al., 2010). The genetic background of the mouse strain seems to be important in this case, because *p21*^−/−^ mice develop lupus in C57BL/6 but are protected from autoimmunity in BXSB strains (Lawson et al., 2004). In humans, SNPs that lower the promoter activity of *P21* are positively associated with lupus nephritis (Kim et al., 2009). An additional cell-cycle-arrest-related gene reported as a lupus susceptibility factor is *GADD45* (Cretu et al., 2009). *Gadd45a*-deficient mice develop a lupus-like syndrome with unrestricted T-cell proliferation (Salvador et al., 2002). Although GADD45A has previously been shown to bind to P21, *Gadd45a*^−/−^*p21*^−/−^ mice develop more severe accelerated disease, suggesting that the two genes play non-redundant roles in the development of SLE (Salvador et al., 2002; Zhao et al., 2000).

### Defective dead-cell clearance in SLE

Apoptotic cells containing nuclear material are a potential source of antigen that can drive TLR activation and autoreactive antibody production in lupus. For example, *c-Mer*-deficient mice display defective phagocytosis of apoptotic cells and, over time, develop autoantibodies against DNA, chromatin and IgG (Cohen et al., 2002). Clearance of chromatin or DNA by endonucleases such as DnaseI is also important in preventing activation of nucleic-acid-induced innate responses. Loss-of-function *DNASE1* mutations and lowered *DNASE1* expression in diseased kidneys of both individuals with SLE and lupus-prone mice have been described (Fenton et al., 2009; Martinez-Valle et al., 2010; Napirei et al., 2000; Seredkina et al., 2009; Tsukumo and Yasutomo, 2004; Zykova et al., 2010). Ineffective DNA clearance might be an aggravating factor at end-stage kidney disease because the increased presence of free nucleic acids might trigger innate sensors locally in tissue (Fenton et al., 2009).

## Prospective treatments for lupus and conclusions

Given the heterogeneous nature of SLE, it has been difficult to design therapies that alleviate all aspects of the disease. Current treatments with steroids aim at total immunomodulation and trigger a variety of side effects when administered on a long-term basis (Liu et al., 2013). The hope is that knowledge from human genetics and mouse models can provide new insights into targets for therapy. These therapies could alleviate chronic inflammation caused by innate pathway activation, reduce the number of autoreactive lymphocytes or minimize target organ destruction. Among the therapies that seem to reduce innate responses during disease, the antimalarial hydrochloroquine is presently being used as a preventive drug in lupus, although the mechanism of prevention is not completely known. Hydrochloroquine increases the pH in intracellular vesicles, and this could act to diminish innate activation through endocytic receptors such as TLRs (Fox, 1993). Inhibitory oligonucleotides that target TLR7 and/or TLR9 seem to be effective in reducing glucocorticoid treatment resistance in lupus backgrounds (Guiducci et al., 2010). Thus, this treatment could potentially act synergistically with the currently recommended steroid regimen. In addition, several active clinical studies are currently testing the efficacy and safety of monoclonal antibody therapies against either IFN-I or the IFN-I receptor in individuals with SLE [topic introduced by Morimoto et al. (Morimoto et al., 2011; reviewed in Lichtman et al., 2012)]. However, the fact that IFN-receptor deficiency only reduces but does not totally eliminate symptoms in murine lupus suggests that IFN blockage might not be completely effective in severe cases of human SLE.

Regarding therapies that target autoreactive lymphocyte activation, blocking T-cell help with CTLA4Ig, an immunosuppressive agent, was successful in mice (Finck et al., 1994), and this approach is now FDA approved for rheumatoid arthritis (Bluestone et al., 2006). Unfortunately, B-cell depletion does not lead to a positive outcome in clinical trials for severe SLE (Davies et al., 2013), and gave mixed results following repeated depletion (Ng et al., 2006). The latest drug approved by the FDA for treatment in lupus is a BlyS-neutralizing antibody that specifically targets B-cell survival (Runkel and Stacey, 2014). This treatment not only reduces autoantibodies but also normalizes the levels of C3 and C4 in patients (Stohl et al., 2012). Drug development for the treatment of lupus has been historically slow, but we are optimistic that all the knowledge gained by human genetics and mechanistic studies in mice will eventually lead to the identification of novel drugs or combinations of them optimally designed for the treatment of SLE.

Overall, the variety of mouse models provides a useful toolbox for the analysis of genetic and environmental factors that affect immune-cell activation and self-reactivity. Perhaps the limitation of current mouse models seems to reside in the lack of models that show a flare-and-wane-type of chronic disease that is most common in human SLE: all mouse models represent severe cases with increased severity of pathology over time. Thus, one of the future goals of mouse models of SLE should be to address the heterogeneity in disease course that characterizes human SLE.
